# Rapid disappearance of acute subdural hematoma due to abrogated hyper-fibrinolytic activity by tranexamic acid: Case report

**DOI:** 10.1097/MD.0000000000035998

**Published:** 2023-11-10

**Authors:** Rong Liu, Yan Li, Seidu A. Richard, Zhigang Lan, Xuesong Liu

**Affiliations:** a Department of Neurosurgery, West China Hospital, Sichuan University, Sichuan, China; b Department of Intensive Care Unit, Luojiang People’s Hospital, Sichuan, China; c Institute of Neuroscience, Third Affiliated Hospital of Zhengzhou University, Zhengzhou, China.

**Keywords:** ASDH, CSDH, D-dimer, hyper-fibrinolysis, TBI, tranexamic acid

## Abstract

**Rationale:**

Acute subdural hematoma (ASDH) occurs after tearing of bridging veins within the dura resulting in the accumulation of blood between the arachnoid and dura layers within 72 hours after traumatic head injury. Also, antigen fibrin D-dimer (DD) is the principal enzymatic degradation product of cross-linked fibrin by plasmin. We observed that early tranexamic acid (TXA) treatment resolved hyper-fibrinolysis and rapid disappearance ASDH.

**Patients concerns:**

A 48-year-old female presented with unconsciousness for 2 hours after head trauma. Her Glasgow Coma Scale score was >8 points.

**Diagnosis:**

Computed tomography scan established ASDH with midline shift and brainstem compression and surgery was scheduled. Also, laboratory results indicated high DD spike of 34,820 μg/L and a reduction in plasma fibrinogen 1 hour after the injury.

**Intervention:**

She was treated with intravenous TXA immediately after admission.

**Outcomes:**

Her DD spike decreased remarkably in 48 hours with associated rapid disappearance of ASDH thereby averting surgical intervention. She recovered fully with no long-term complications.

**Lessons:**

Historically, hyper-fibrinolysis is associated with poor outcome in head trauma. However, early initiation of TXA which is noninvasive treatment modality for ASDH could avert surgery and reduce cost, anesthesia, and other complications associated with surgery.

## 1. Introduction

Acute subdural hematoma (ASDH) occurs after tearing of bridging veins within the dura resulting in the accumulation of blood between the arachnoid and dura layers within 72 hours after traumatic brain injury (TBI).^[1,2]^ The aged are more prone to subdural hematomas (SDHs) after traumatic injuries.^[3,4]^ Currently, the prevalence SDH range from 1.7 to 20.6 per 100,000 people worldwide.^[3]^ ASDH often progress into subacute hematomas and finally chronic subdural hematomas (CSDH) if early interventions are not initiated.^[4,5]^ The gold standard radiological modality for the detection of all forms of SDH is computed tomography (CT-scan) after head injury.^[6–8]^ Although medical management with corticosteroids has proven to effective, surgery still remain the standard treatment modality for all forms SDH base on CT-scan parameters such as thickness and midline shift.^[9]^

Antifibrinolytic agents like e-aminocaproic acid as well as tranexamic acid (TXA) have been utilized to decrease hemorrhage after severe trauma.^[10]^ These agents lessen blood loss by blocking the ability of plasmin to degrade fibrin resulting in a reduction in clot dissolution.^[11]^ Also, it is known that antigen fibrin D-dimer (DD) is the principal enzymatic degradation product of cross-linked fibrin by plasmin.^[12]^ Systemic concentration of DD is key index of fibrin turnover in the blood and a key indicator of fibrinolytic status.^[12]^ Almost all studies on the use of TXA for the treatment of subdural hematoma focused on CSDH and not the ASDH.^[6–8]^ We observed that early TXA treatment resolved hyper-fibrinolysis and rapid disappearance ASDH.

## 2. Case presentation

A 48-year-old female presented with unconsciousness for 2 hours after head trauma. Physical examination revealed a swelling on the right side of the head and a scalp laceration about 5 cm long which reached deep into the skull with active bleeding. Her eyelids were swollen and bluish with a diameter of about 0.3 cm on both sides and slow to light and touch reflex. Her neck was stiff indicting meningeal irritation. Also, Kernigs sign and Brudzinski sign were negative. We observed no eye opening to pressure, no response to sounds and she could only localize. Thus, her Glasgow Coma Scale (GCS) score was > 8 points.

**Figure 1.**
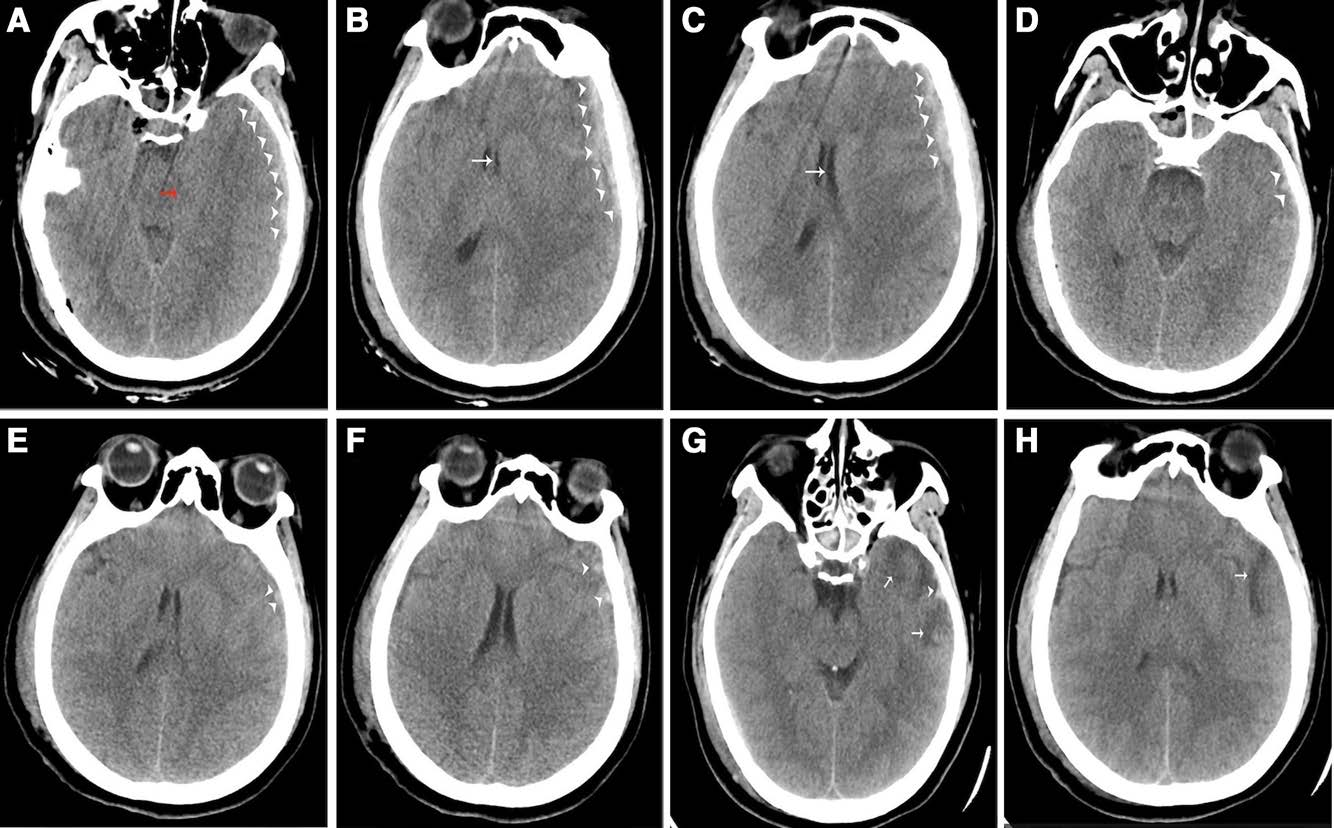
CT images taken during admission, 4 hours after admission, and 72 hours after admission. (A–C) CT 1 hour after trauma revealed left temporal and frontal subdural hematoma (arrowheads), brainstem compression (red arrow), and midline shift (white arrows). (D–F) CT 4 hours later showed disappearing subdural hematoma (arrowheads), healthy ambient cistern, and restored midline. (G and H) CT 72 hours later showed the barely visible subdural hematoma (arrowhead) and slight edema (white arrows).

Brain computed tomography (CT) scan done immediately after arrival in the hospital revealed an ASDH with associated midline shift and brainstem compression (Fig. 1A–C). A surgical plan of hematoma evacuation and decompressive craniectomy was made. However, she spontaneously opened her eyes, and responded to all verbal commands but she was however confused. Thus, her GCS changed to 14 while waiting for surgery. A repeated CT scan done 4 hours after trauma showed rapid absorption of the ASDH and restoration of the midline (Fig. 1D–F). The surgery was canceled. A third CT scan done 72 hours after the trauma revealed further absorption of the ASDH (Fig. 1G and H).

A review of the patient’s records revealed she was treated with intravenous TXA 10 mls:0.1 g, a total dose of 0.3 g immediately after admission prior to CT scan and managed as an unconsciousness patient as per our department protocols. She was not given corticosteroids. Also, a review of her coagulatory laboratory results (Table 1) revealed normal rangers. However, a DD spike of 34,820 μg/L with a reduction in plasma fibrinogen 1 hour after the injury and decreased to 1240 μg/L in 48 hours and increase again to 4490 μg/L a week later. Blood gases levels were at normal rangers from admission to discharge from hospital.

**Table 1:** The coagulation profile of the patients on arrival at hospital, 48 hours in admission and 1 week in admission.

Marker/time	Arrival at hospital	48 hours	1 week	Normal range
Thrombin time (PT)	12.60	12.00	11.60	12–14 s
Prothrombin ratio (INR)	1.10	1.04	1.01	0.8–1.1
Activated partial prothrombin time	26.80	29.10	26.00	21–35 s
Plasma fibrinogen (Fib)	**1.50**	**3.35**	**2.82**	2.0–4.0 g/L
Thrombin time assay (TT)	14.30	12.40	14.90	12–19 s
Procalcitonin (PCT)	0.04	0.10	0.05	<0.1 ng/mL
**D-Dimer (DD**)	**34,820.00**	**1240.00**	**4490.00**	<250 ng/mL
White blood cell count (WBC)	19.58	10.10	7.78	4.5–11.0 × 10^9^/L
Platelet count (PLT)	215	128	386	150–400 × 10^9^/L
Mean platelet volume (MPV)	11.60	14.10	10.90	7–9 fL
Platelet distribution width (PDW)	16.00	18.60	13.20	8.3%–56.6%
Plateletcrit (PCT)	0.25	0.18	0.42	0.22%–0.24%
Platelet larger cell ratio (P-LCR)	41.90	51.40	28.80	15%–35%

She received conservative treatment afterwards as per our hospital protocols and was discharged 2 weeks later. She recovered fully with no long-term complications. Six months follow-up revealed no recurrence of the lesion and she is well and goes about her normal daily activities.

## 3. Discussion

SDH occurs when is blood accumulated in between the arachnoid and dura layers after TBI.^[1,2,4,5]^ This pathological occurrence is often classified into acute, subacute, or chronic.^[1,2]^ ASDH often occurs within 72 hours after head injury while subacute hematomas occur within 4 to 21 days of a head injury and CSDH occur 21 days after a head injury.^[13]^ Hyper-fibrinolytic activities are very crucial in the liquefaction as well as advancement of the hematoma from acute of chronic.^[6]^ We believed hyper-fibrinolysis triggered the enlargement and liquefaction of ASDH in our patient and early administration if TXA averted her symptomatology as well as rapid disappearance of the ASDH.

Studies on the use of TXA for the treatment of SDH focused on CSDH^[6–8]^ with no report on the use of TXA in the treatment of ASDH or prophylaxis for subdural hematoma. We observed that early TXA treatment resolved hyper-fibrinolysis and rapid disappearance ASDH. The hemorrhagic diathesis related to hyper-fibrinolysis has a critical impact on the mortality as well as functional outcome of patients TBI.^[14]^ Hypercoagulation peaked immediately after injury, fibrinolysis peaked 3 hours after injury, and fibrinolysis shutdown peaked 6 hours after injury which are characterize with poor long-term outcomes after TBI.^[14]^ It is worth noting that hyperfibrinolysis is associated with poor outcome in head trauma.^[14]^

It is worth noting that surgery still remain the standard treatment modality for all forms SDH base on CT-scan parameters like thickness and midline shift. Surgical intervention allows for substantial amelioration of symptomatology as well as brain decompression.^[1,9]^ It is well established that SDH with a thickness of >10 mm or midline shift >5 mm on CT scan necessitates surgical intervention regardless of GCS as stipulated by the Brain Trauma Foundation.^[1,9]^ Furthermore, patients presenting with GCS <9 and SDH thickness >10 mm as well as midline shift <5 mm necessitates surgical intervention if the GCS declined by 2 or more points in a definite time range, changes in symptomology, or with an intracranial pressure > 20 mm Hg.^[9,14]^

CT scan done immediately after the arrival of our patient in the hospital revealed an ASDH, midline shift and brainstem compression which are cardinally indication of surgical intervention. However, she spontaneously opened her eyes, and responded to all verbal commands but she was however confused indicating that her GCS changed to 14 while waiting surgery. A repeated CT scan showed rapid absorption of the ASDH and restoration of the midline, thus, the surgery was canceled. Corticosteroids which have been implicated in the blockade of the synthesis of several proinflammatory mediators, immune system cells, proinflammatory enzymes, as well as the synthesis of nitric oxide and cyclooxygenase have been proposed as medical treatment because they are able to reduce inflammation as well as angiogenesis in mild CSDH patients.^[15,16]^

It is worth noting that our patient did not receive corticosteroids therapy. TXA is a precise antifibrinolytic agent that inhibits plasminogen stimulation as well as plasmin activity.^[6,17]^ It is a derivative of the amino acid lysine which exerts antifibrinolytic effects via reversibly binding to lysine sites on plasminogen resulting in the inactivation of plasminogen.^[5,11,17]^ Also, it is well known that antigen fibrin DD is the principal enzymatic degradation product of cross-linked fibrin by plasmin. Systemic concentration of DD is key index of fibrin turnover in the blood and a key indicator of fibrinolytic status. Several studies have reported the successful usage of TXA to treat patients with CSDH with no recurrence.^[6–8]^

The patient was treated with intravenous TXA immediately after admission and managed as an unconsciousness patient per of department protocols. We observed a DD spike with a reduction in plasma fibrinogen 1 hour after the injury and decreased remarkably in 48 hours and increase again a week later. We believe early TXA treatment resolved hyper-fibrinolysis and rapid disappearance ASDH thereby averting surgical intervention in our patient. She recovered full with no long-term complications and no recurrence of the SDH.

## 4. Conclusion

The utilization of TXA for the treatment of SDH focused on CSDH with no reports on its usage in the treatment of ASDH or prophylaxis for SDH. Early initiation of TXA which is noninvasive treatment modality for ASDH could avert surgery and reduce cost, anesthesia and other complications associated with surgery.

## Acknowledgments

The authors thank the patient’s trustee for granting permission to publish his case.

## Author contributions

**Conceptualization:** Rong Liu, Yan Li, Seidu A Richard, Zhigang Lan, Xuesong Liu.

**Data curation:** Rong Liu, Yan Li, Seidu A Richard, Zhigang Lan, Xuesong Liu.

**Formal analysis:** Yan Li, Seidu A. Richard, Zhigang Lan, Xuesong Liu.

**Investigation:** Rong Liu, Zhigang Lan, Xuesong Liu.

**Methodology:** Rong Liu, Yan Li, Seidu A Richard, Zhigang Lan, Xuesong Liu.

**Resources:** Zhigang Lan, Xuesong Liu.

**Validation:** Zhigang Lan, Xuesong Liu.

**Writing – original draft:** Seidu A. Richard.

**Writing – review & editing:** Rong Liu, Yan Li, Seidu A. Richard, Zhigang Lan, Xuesong Liu.
